# Multimode Friedel oscillations in monolayer and bilayer graphene

**DOI:** 10.1038/s41598-024-63738-w

**Published:** 2024-06-14

**Authors:** Chiun-Yan Lin, Chih-Wei Chiu

**Affiliations:** 1 Department of Physics, National Cheng Kung University, Tainan, Taiwan; 2 Department of Physics, National Kaohsiung Normal University, Kaohsiung, Taiwan

**Keywords:** Monolayer graphene, Bilayer graphene, Friedel oscillations, Electronic properties and materials, Surfaces, interfaces and thin films

## Abstract

This study systematically explores the influence of charged impurities on static screening in monolayer graphene and extends the investigation to AA-stacked and AB-stacked bilayer graphene (BLG). Applying the random phase approximation (RPA), monolayer graphene displays unique beating Friedel oscillations (FOs) in inter-valley and intra-valley channels. Shifting to BLG, the study emphasizes layer-specific responses on each layer by considering self-consistent field interactions between layers. It also explores the derived multimode FOs, elucidating distinctions from monolayer behavior. In AA-stacked BLG, distinct metallic screening behaviors are revealed, uncovering unique oscillatory patterns in induced charge density, providing insights into static Coulomb scattering effects between two Dirac cones. The exploration extends to AB-stacked BLG, unveiling layer-specific responses of parabolic bands in multimode FOs with increasing Fermi energy. This comprehensive investigation, integrating RPA considerations, significantly advances our understanding of layer-dependent static screening in the broader context of FOs in graphene, providing valuable contributions to the field of condensed matter physics.

## Introduction

Exploring quantum phenomena in two-dimensional (2D) materials unveils a unique platform for understanding subatomic-scale interactions. This study delves into Friedel oscillations (FO) in graphene systems, intricate charge density ripples initially elucidated by J. Friedel in metallic materials^1^. FOs are pivotal for investigating how charge density responds to impurities, involving fermions in a Fermi gas^2^. Unlike classical electric charge screening, these oscillations demand a quasi-particle or scattering treatment for precise description^3^. Impurity interactions with carriers introduce static screening behavior, mediated by long-range interactions between charges. The wave vector *k* concerning the Fermi surface is pivotal in understanding FOs’ manifestation and their correlation with the dielectric polarizability in 2D systems^4^. FOs, stemming from long-range Coulomb interaction, manifest as a quantum phenomenon driven by the singularity at the Fermi wave vector, improving the static limit in understanding FO origins.

In a broader context, graphene stands out among emerging materials as exhibiting a unique Coulomb response^5–11^. The introduction of doping in few-layer graphene adds complexity to static charge screening, involving intra-layer and inter-layer Coulomb interactions, which paints a nuanced picture of static electric polarizations^5,6^. Scanning tunneling microscopy (STM) emerges as a powerful tool for observing FOs. STM images provide insights into the quasiparticle interference of FOs within the local density of states. They reveal that efficient scattering processes rely on intra-valley and inter-valley scattering on the Fermi surface driven by the Coulomb field in graphene^12–16^, showcasing their relevance across various underlying topological properties^17–19^. Furthermore, STM images depend on the Fermi surface contour, and their Fourier transform (FT) map unveils spatial standing waves induced by electronic quasiparticle interference at edges or stacking boundaries, as observed in monolayer graphene^20,21^, BLG^22^, and rhombohedral trilayer graphene^23^.

FOs in 2D materials play a crucial role in unraveling complex quantum interactions, contributing to a deeper understanding of the physics underlying FOs within 2D systems. This work introduces a theoretical framework for reliably calculating the dielectric function, emphasizing the static limit and employing FT to examine the spatial FO characteristics. The investigated systems include monolayer graphene, AA-stacked bilayer graphene (BLG), and AB-stacked BLG, revealing layer-specific attributes and stacking-dependent FO behaviors. Employing a model with layer-based tight-binding functions, it integrates response functions for a self-consistent field approach, systematically investigating dominant electron scattering mechanisms, effective screening length, and long-range charge oscillation. This facilitates real-space observation of FOs through high-resolution STM experiments^20–23^.

The interplay between valley scattering is distinctly crucial in graphene systems. Theoretical predictions utilizing the T-matrix approximation have extensively analyzed static scattering from localized impurities in monolayer graphene^14,15^, AB-stacked BLG^14,15^, and rhombohedral graphene^16^. The random phase approximation (RPA) has primarily been applied to monolayer graphene with intra-valley scattering^9^. However, these analyses often focus on the Fermi surface contours derived from the first pair of subbands nearest to the Fermi level. In this study, we utilize a modified RPA framework that integrates complete charge screening from all pairs of energy bands across multiple Fermi contours, revealing multimode behaviors with increasing electron doping.

It should be noticed that beat patterns in the electron density profile have been verified as a result of valley scattering on multiple Fermi circles by graphene armchair and zigzag edges^20^. This study also reveals unique beating FOs that consider both inter-valley and intra-valley channels simultaneously. Moreover, the presence of multi-critical momentum in BLG provides insights into essential quasiparticle properties related to stacking-dependent and valley-mixing charge screening among layers. The anticipation of charge screening phenomena extends to other emergent 2D layered materials with two inequivalent valleys, such as silicene^24^, germanene^25^, and tinene^26^, including scenarios where the anisotropic Fermi surface is induced by uniaxial strains^27^ or adatom chemisorption^28^. Furthermore, the resonance of charge screening phenomena heralds a promising future for discoveries and technological advancements in these diverse 2D layered materials.

## Methods: theoretical framework of static response function and Friedel oscillations

The theoretical framework developed here aims to explore the static response and resulting FOs in materials. Utilizing the self-consistent Dyson formula, we evaluate screened Coulomb potentials and induced charge densities, accounting for the dominant electron scatterings arising from intra-valley and inter-valley transitions in graphene. This study focuses on 2D FOs in graphene, offering insights into charge density behaviors influenced by impurities and defects. Moreover, we conduct a comprehensive analysis of electron correlations in layered graphene, highlighting the role of $\pi$ electrons in responding to Coulomb perturbations. By utilizing tight-binding schemes, one can extensively calculate elementary excitations, Coulomb decay rates, and plasmon excitations^6,10,11^. This self-consistent Dyson formula straightforwardly aids in unraveling the mechanisms underlying static electron scatterings, shedding light on impurity effects in layered graphene.

Graphene consists of tightly bound carbon atoms in a hexagonal honeycomb lattice, with $\pi$ orbitals describing its unique electronic properties. With two primitive carbons in each graphene sheet, a total of $2\times N_{l}$ atoms is obtained for the $N_{l}$-layered system. The tight-binding Hamiltonian is formulated using the 2p$_z$ orbitals in a primitive unit cell to capture the essential properties of graphene near its inequivalent valleys (*K* and $K^{\prime }$). This is expressed as:

1 $$\begin{aligned} H=-\sum _{i,j}\gamma _{ij}c_{i}^{\dag }c_{j}\text {.} \end{aligned}$$

Here, $\gamma _{ij}$ represents the hopping integral between the *i*-th and *j*-th lattice sites, and $c_{i}^{\dag }$ and $c_{j}$ are the creation and annihilation operators on the lattice sites. This approach has been successfully employed to calculate the energy dispersions of various sp$^2$-bonding graphenes with different stacking symmetries^29^. It is evident that the representative hopping terms depend on the geometric structures, encompassing those describing monolayer, AA-stacked, and AB-stacked BLG, as well as various trilayer or tetralayer graphenes, under the interplay with external magnetic fields^29^ and electron-electron interactions^6,10,11^.

When a charged impurity is implanted on a graphene sheet, it induces fluctuations in charge density on each graphene layer. The charge redistributions result in the screening of Coulomb potentials on the graphene layers, described by the dimensionless dielectric function $\epsilon (q)$. Within the linear response regime, $\epsilon (q)$, defined as the ratio between the external potential and the effective potential, is given by

2 $$\begin{aligned} \epsilon ({\textbf{q}})=\lim _{V^{ex}\rightarrow 0}\frac{V^{ex}({\textbf{q}})}{V^{eff}({\textbf{q}})}\text {.} \end{aligned}$$

This function can also be characterized by the charge densities and the longitudinal electric fields. Importantly, $\pi$ electrons play a key role in static charge screenings and respond directly to Coulomb perturbations, while $\sigma$ electrons contributes to the background dielectric constant $\epsilon _0$, evaluated at 2.4 based on reflectance measurements^7^.

In $N_{l}$-layer graphene, the dielectric function is represented as a $N_{l}\times N_{l}$ tensor within a lattice-based RPA framework^10,11^:

3 $$\begin{aligned} \epsilon _{ll^{\prime }}({\textbf{q}})=\epsilon _{0}\delta _{ll^{\prime }}- \sum ^{N_{l}}\limits _{m=1}V_{lm}({\textbf{q}})\chi _{m,l^{\prime }}({\textbf{q}})\text {,} \end{aligned}$$

where *l* and $l^{\prime }$ denote the layer index and $\delta _{ll^{\prime }}$ is the Kronecker delta function. $\chi _{m,l^{\prime }}$ represents the bare polarization function, serving as the linear coefficient in the response theory, providing unprecedented understanding of charge density behavior and insights into the world of 2D FOs in the materials^3^. Finally, the bare Coulomb potential tensor $V_{ll}^{\prime }$ is expressed as $v_{q}e^{-q|l-l^{\prime }|d_{0}}$, in line with the characteristics of a 2D electron gas, where $v_q=2\pi e^2/q$.

In the expansion of the $N_{l}\times N_{l}$ response tensor, we have:

4 $$\begin{aligned} \chi _{m,l^{\prime }}({\textbf{q}})= 2\sum \limits _{k}\sum \limits _{n^{c}n^{v}}&|_{m}\langle {n^{v},{\textbf{k}}}| e^{-i{\textbf{q}}\cdot {\textbf{r}}}|n^{c},\mathbf {k+q}\rangle _{l^{\prime }}|^{2} \times \frac{f(E^{n^{v}}({\textbf{k}}))-f(E^{n^{c}}(\mathbf {k+q}))}{E^{n^{v}}({\textbf{k}})-E^{n^{c}}(\mathbf {k+q})}\text {,} \end{aligned}$$

where electronic scattering is considered across the entire Brillouin zone. The prefactor 2 accounts for spin degeneracy, and the Fermi-Dirac distribution function is given by $f(E^{n^{c,v}}({\textbf{k}}))=1/[1+\exp (E^{n^{c,v}}({\textbf{k}})-\mu (T))/k_{B}T]$, where $k_{B}T$ and $\mu (T)$ represent the product of Boltzmann const with temperature and chemical potential, respectively. Also, Eq. (4) encompasses the entirety of charge screening between the initial and final states, denoted as $E^{n^{v}}({\textbf{k}})$ and $E^{n^{c}}(\mathbf {k+q})$ respectively, where the subscript $n^{c}$ ($n^{v}$) indicates the valence (conduction) band index. These terms are distinguished as intralayer responses when $m=l^{\prime }$ and interlayer responses when $m\ne l^{\prime }$ under static Coulomb interactions. Furthermore, the expectation value of the electron-electron interaction in Eq. (4) is calculated in the tight-binding scheme:

$$\begin{aligned} |&_{m}\langle {n^{v},{\textbf{k}}}| e^{-i{\textbf{q}}\cdot {\textbf{r}}}|n^{c},\mathbf {k+q}\rangle _{l^{\prime }}|^{2}= \\&I(q)\int \limits _{1stBZ}\frac{d{\textbf{k}}^{2}}{(2\pi )^{2}} \biggl ( \widetilde{U_{m}}^{*}(n^{v},{\textbf{k}})\widetilde{U_{l^{\prime }}}(n^{c},\mathbf {k+q})\biggr ) \biggl ( \widetilde{U_{m}}(n^{v},{\textbf{k}})\widetilde{U_{l^{\prime }}}^{*}(n^{c},\mathbf {k+q})\biggr )\text {.} \end{aligned}$$

Here, ${\widetilde{U}}\widetilde{U^{*}}$ represents the inner product of two Bloch functions, and *I*(*q*) calculated from $\pi$ orbitals is crucial in inter-valley transitions (for the explicit expression, see^7^). To consider the effects of band structure, the layer-decomposed wave functions scheme is employed.

In the self-consistent RPA consideration, the effective Coulomb potential $V_{eff}$ under the external potential $V_{ex}$ satisfies the tensor equation below, in terms of the dielectric tensor in Eq. (3):

5 $$\begin{aligned} \epsilon _{0}V^{eff}_{ll^{\prime }}({\textbf{q}},\omega )=V^{ex}_{ll^{\prime }}({\textbf{q}})+ \sum \limits _{mm^{\prime }}V^{ex}_{lm}({\textbf{q}})\chi _{mm^{\prime }}({\textbf{q}},\omega ) V^{eff}_{m^{\prime }l^{\prime }}({\textbf{q}},\omega )\text {.} \end{aligned}$$

The second term, representing the induced potential, is correlated with $V_{eff}$ within the self-consistent approach. This indicates that the static screening in BLG, probed by electrons across its layers, integrates effective contributions from all layers into the induced potential on a single layer. Alternatively, the induced charge density is given by:

6 $$\begin{aligned} N^{in}_{ll^{\prime }}({\textbf{q}})=\sum ^{N_{l}}\limits _{m=1}V^{eff}_{lm}({\textbf{q}})\chi _{m,l^{\prime }}({\textbf{q}})\text {.} \end{aligned}$$

Both $V^{eff}({\textbf{q}})$ and $N^{in}({\textbf{q}})$, resulting from the available scattering channels across different graphene layers, can be realized. High-resolution STM measurements allow the observation and characterization of intra-valley and inter-valley scatterings in graphene^12^. The verification offers insights into the electronic behavior at various Fermi surface contours within its Brillouin zone. Therefore, these derived equations facilitate studies on screening effects in layered graphene caused by desired impurities.

In this study, our primary focus is on 2D graphene, specifically in monolayers, as well as AA- and AB-stacked BLG. We employ inverse FT to derive real-space FOs. The spatial effective potential and induced charge tensors are expressed as follows:

7 $$\begin{aligned} V^{eff}_{ll^{\prime }}({\textbf{r}}) =\sum _{{\textbf{q}}} V^{eff}_{ll^{\prime }}({\textbf{q}}) \exp (i{\textbf{q}}\cdot {\textbf{r}})\text {,} \end{aligned}$$

and

8 $$\begin{aligned} N^{in}_{ll^{\prime }}({\textbf{r}}) =\sum _{{\textbf{q}}} N^{in}_{ll^{\prime }}({\textbf{q}}) \exp (i{\textbf{q}}\cdot {\textbf{r}})\text {,} \end{aligned}$$

where the summation is replaced by an integral in the Brillouin zone. Hereafter, for simplicity, we omit the superscript in $N^{in}_{ll^{\prime }}({\textbf{r}})$, as we are only discussing induced densities in real space within this context.

This work explores a spectrum of factors influenced by impurities under RPA, including the intricate interplay of 2$p_{z}$ orbital hybridizations and Coulomb interactions in graphene. Particularly, the interlayer and intralayer components are straightforwardly examined within a layer-based framework. Electron scatterings, screening length, and the mechanisms behind long-range charge oscillations are thoroughly discussed. The behavior of the dielectric function concerning the Fermi surface is highlighted in relation to the manifestation of FOs. Two scenarios of intra-valley and inter-valley scatterings are investigated, with their correlation with different Fermi surfaces in BLG causing oscillations of multiple modes, influenced by the charge doping concentration. High-resolution STM can be utilized for direct observation of phenomena arising from the static Coulomb field, providing insights into the underlying physics of these materials.

## Results and discussion

The essence of FOs stems from the singularity of the dielectric function at the critical momentum $q_{c}$, reflecting the discontinuity within the Fermi-Dirac distribution at the Fermi level. Graphene showcases distinctly non-analytical characteristics in $\chi (q)$, unlike the 2D electron gas, contingent upon its doping level. Investigations on monolayer graphene offer valuable insights into FOs, influenced by electron doping effects, charge rearrangements in different valleys, and providing a glimpse into altered screening effects and varied oscillations.

This inquiry establishes a foundation for comprehending screening phenomena in graphene systems. Bilayer graphene, with AA and AB stacking configurations, provides an intriguing platform for exploring complex multimode FOs. Effective screening, achievable through the RPA self-consistent field approach, enhances the investigation. Unique stacking symmetries and electronic properties lead to diverse oscillation modes, necessitating a thorough exploration of their intricate behaviors in few-layer graphene. This section aims to elucidate the distinctive attributes of these materials, encompassing both intralayer and interlayer screening, while emphasizing stacking-dependent multimode behaviors on different layers.

### Beating FO in monolayer graphene: impact of doping on charge screening

**Figure 1:**
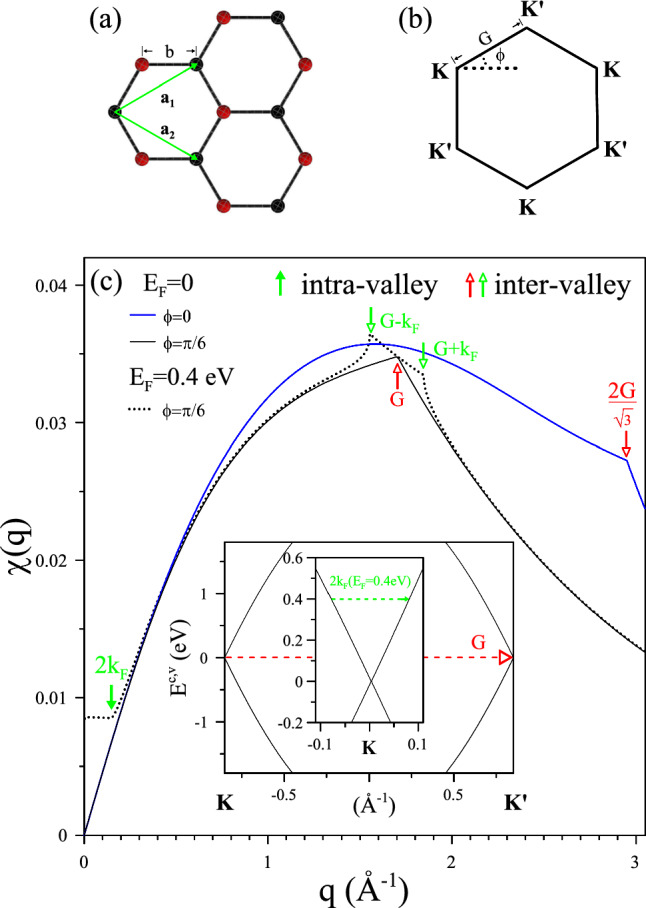
Structural and electronic characterization of monolayer graphene: (**a**) Hexagonal lattices of a monolayer graphene sheet with two primitive vectors, $a_{1}$ and $a_{2}$. The red and black atoms denote A and B atoms, respectively, and $b=1.42$ ${\text{\AA }}$ represents the bond length. (**b**) Brillouin zone featuring *K* and $K^{\prime }$ valleys. (**c**) The static polarization function $\chi (q)$ of monolayer graphene at $E_{F}=0$ and $E_{F}=0.4$ eV. The non-analytic behavior of $\chi (q)$ due to intra-valley and inter-valley transitions is indicated by solid and hollow arrows, with red and green colors corresponding to $E_{F}=0$ and $E_{F}=0.4$ eV, respectively. Inserts depict the corresponding transitions in the band structure.

**Figure 2:**
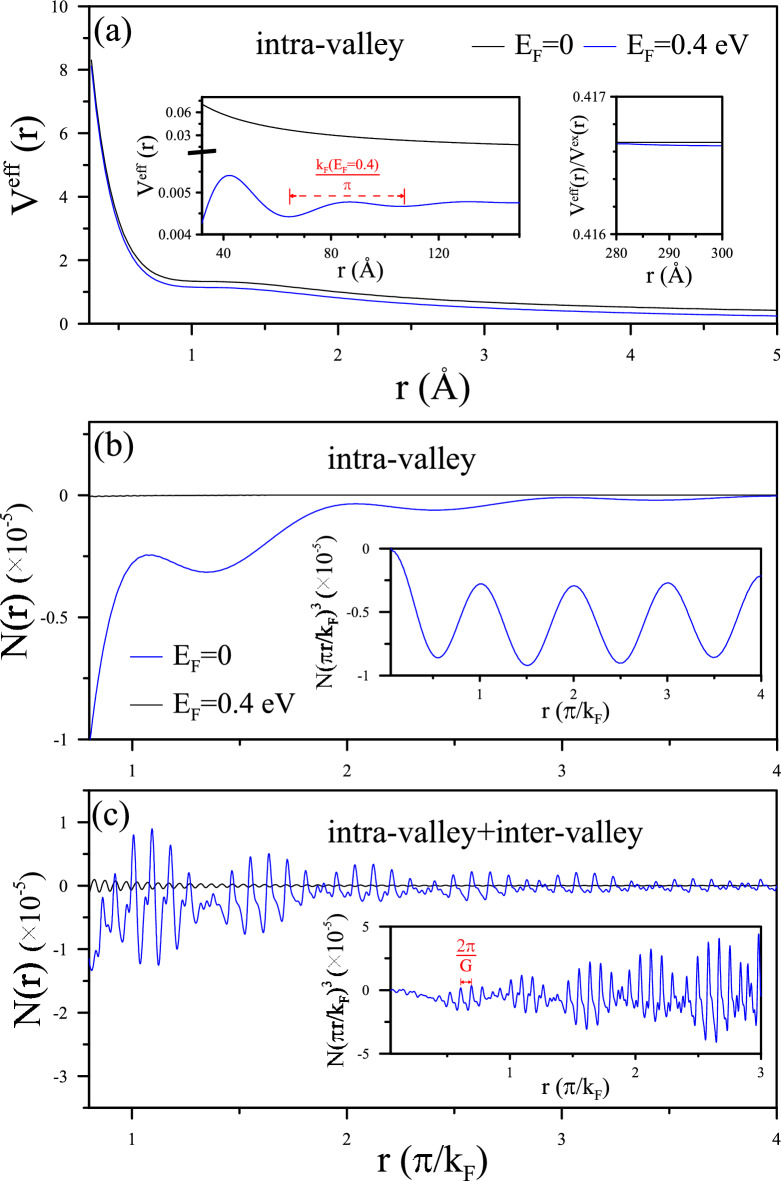
Effective Coulomb potential and induced charge density in monolayer graphene: (**a**) Effective Coulomb potential $V^{eff}(r)$ of monolayer graphene at $E_{F}=0$ and $E_{F}=0.4$ eV induced by a charged impurity at $r=0$. The left inset exhibits scaled FOs by $\pi /k_{F}$, while the right inset illustrates the asymptotic behavior of $V^{eff}(r)$. (**b**) Intra-valley, (**c**) inter-valley (K-K$^\prime$), and intra-valley scatterings contributing to the induced charge density *N*(*r*), featuring an insert highlighting the oscillation period of $\pi /k_{F}$ and a $r^{-3}$ decay.

**Figure 3:**
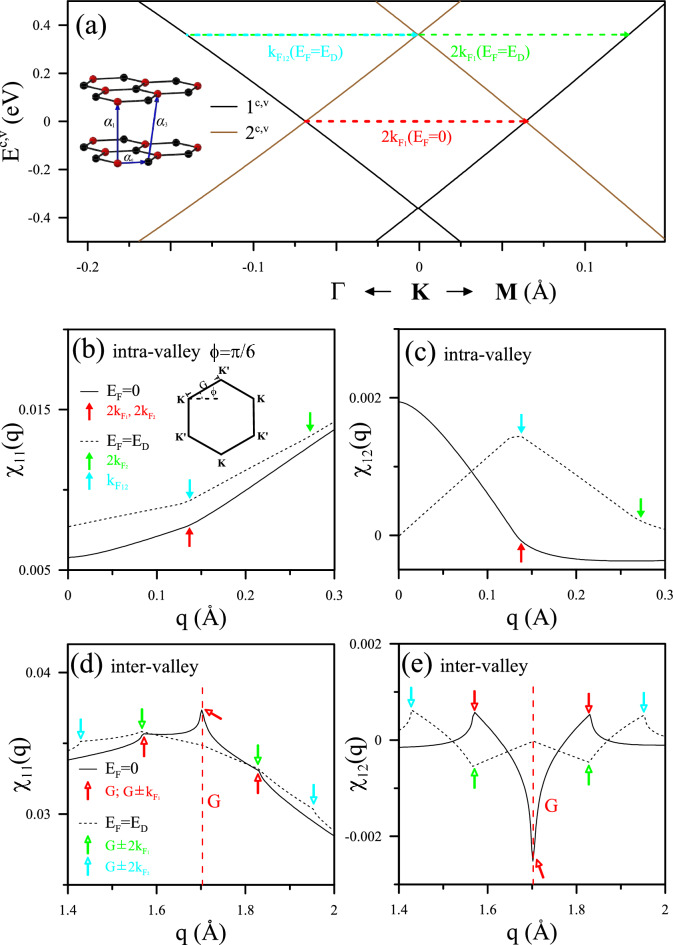
Analysis of band structure and scattering phenomena in AA-stacked bilayer graphene: (**a**) Band structure of AA-stacked BLG along $\Gamma -K-M$, featuring indicated hopping parameters in the left inset. The analytic behavior of intra-valley scattering is illustrated at $\phi =\pi /6$ for (**b**) $\chi _{11}(q)$ and (**c**) $\chi _{12}(q)$ at $E_{F}=0$ and $E_{F}=E_{D}=\alpha _{1}$. Colored solid and hollow arrows emphasize the critical momentum $q_{c}$ governing the scattering on the two Fermi circles. Corresponding plots for inter-valley ($K-K^{\prime }$) scattering are shown in (**d**) and (**e**).

**Figure 4:**
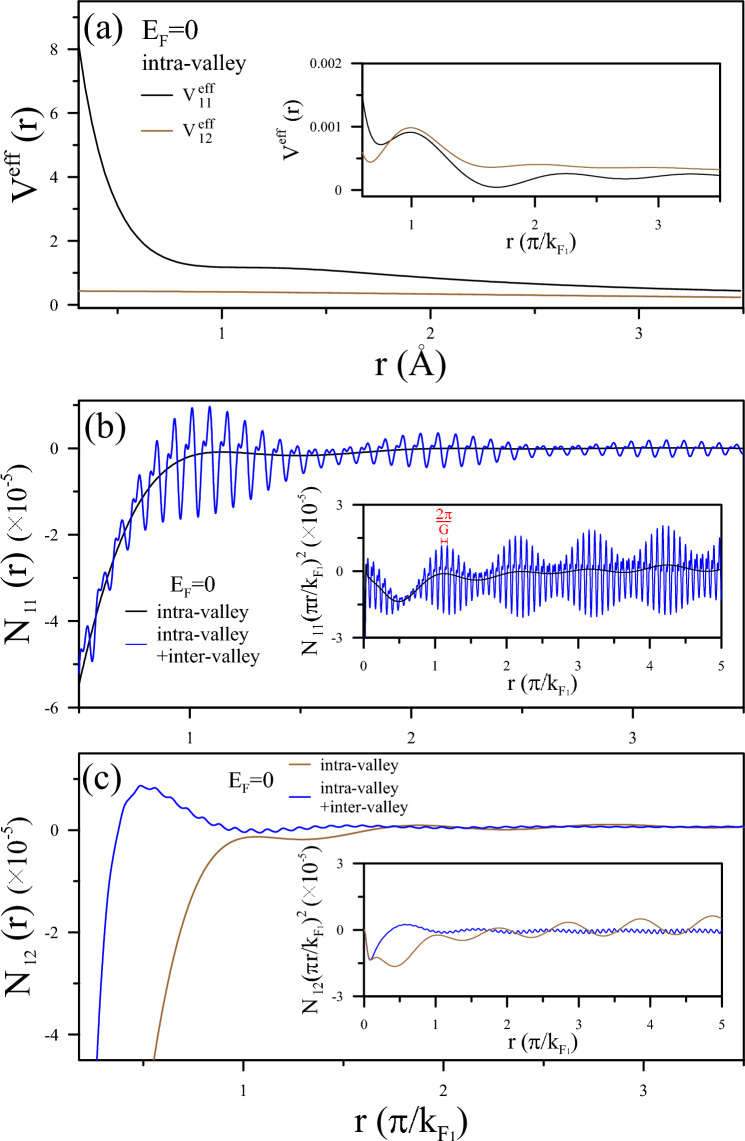
Analysis of effective potentials and induced charge density in AA-stacked BLG: (**a**) Effective potentials $V^{eff}_{11}$ and $V^{eff}_{12}$ for an AA-stacked BLG due to intra-valley scattering at $E^{F}=0$ eV. A charged impurity is located at $r=0$ in the first layer. The inserts display $V^{eff}_{11}$ and $V^{eff}_{12}$, representing a period of $\pi /k_{F_{1}}$ at long distances. (**b**) and (**c**) illustrate the induced charge density $N^{in}_{11}$ and $N^{in}_{12}$ contributed by intra-valley and inter-valley scattering.

**Figure 5:**
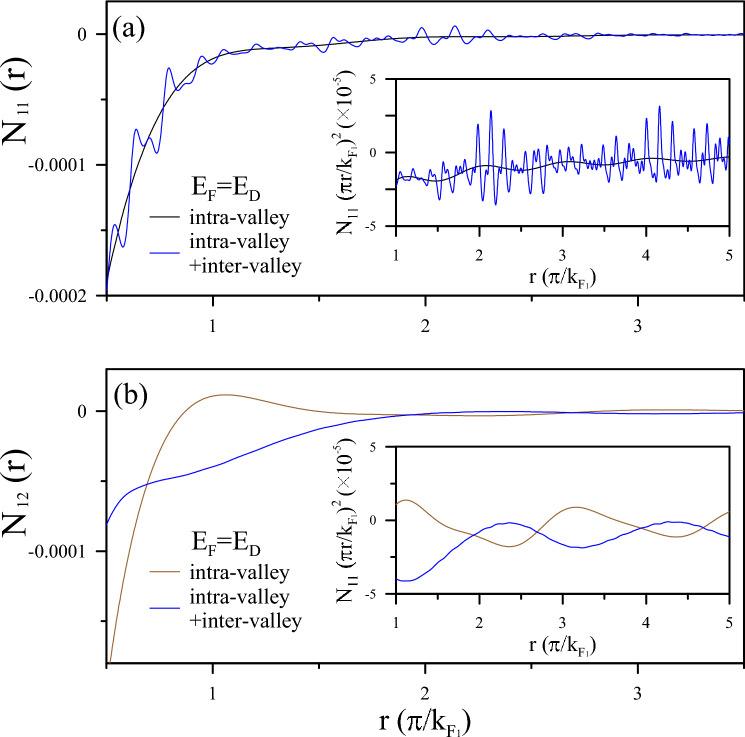
Multimodal FOs in AA-stacked BLG: Presented are the multimodal FOs in AA-stacked BLG. Panels (**a**) and (**b**) illustrate the oscillating $N^{in}_{11}$ and $N^{in}_{12}$ contributed by intra-valley and inter-valley scattering at $E_{F}=E_{D}=\alpha _{1}$. The x-coordinate is scaled by $k_{F_{1}}$ under the various Fermi levels.

**Figure 6:**
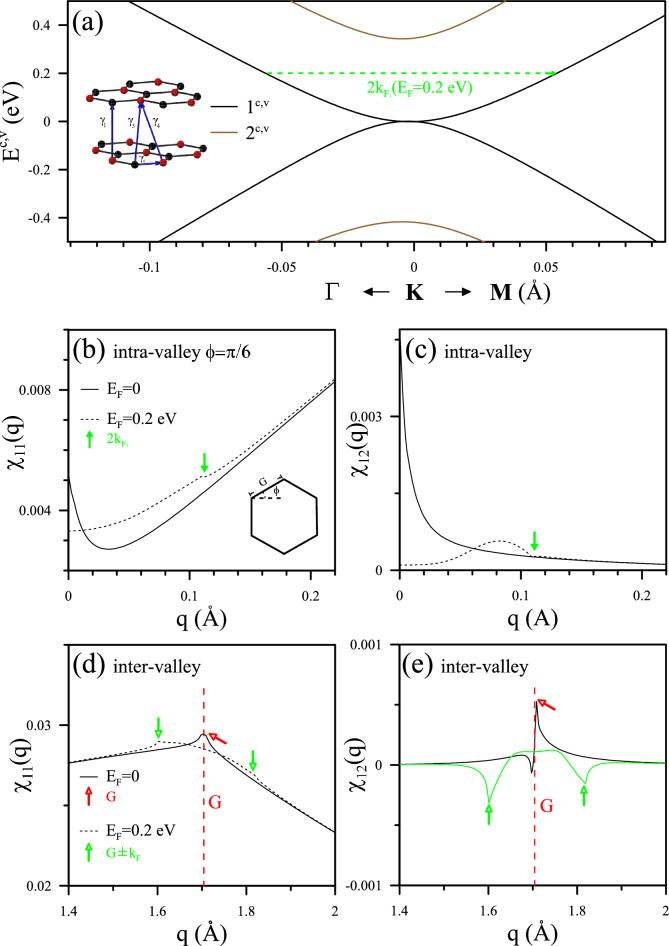
Comparative plot for AB-stacked BLG: Figure depicting (**a**) the geometric structure, hopping parameters $\gamma$, and band structure of AB-stacked BLG, alongside (**b**)–(**e**) the corresponding static polarization functions $\chi _{11}(q)$ and $\chi _{12}(q)$. Colored solid and hollow arrows highlight the critical momentum $q_{c}$ for each scattering process.

**Figure 7:**
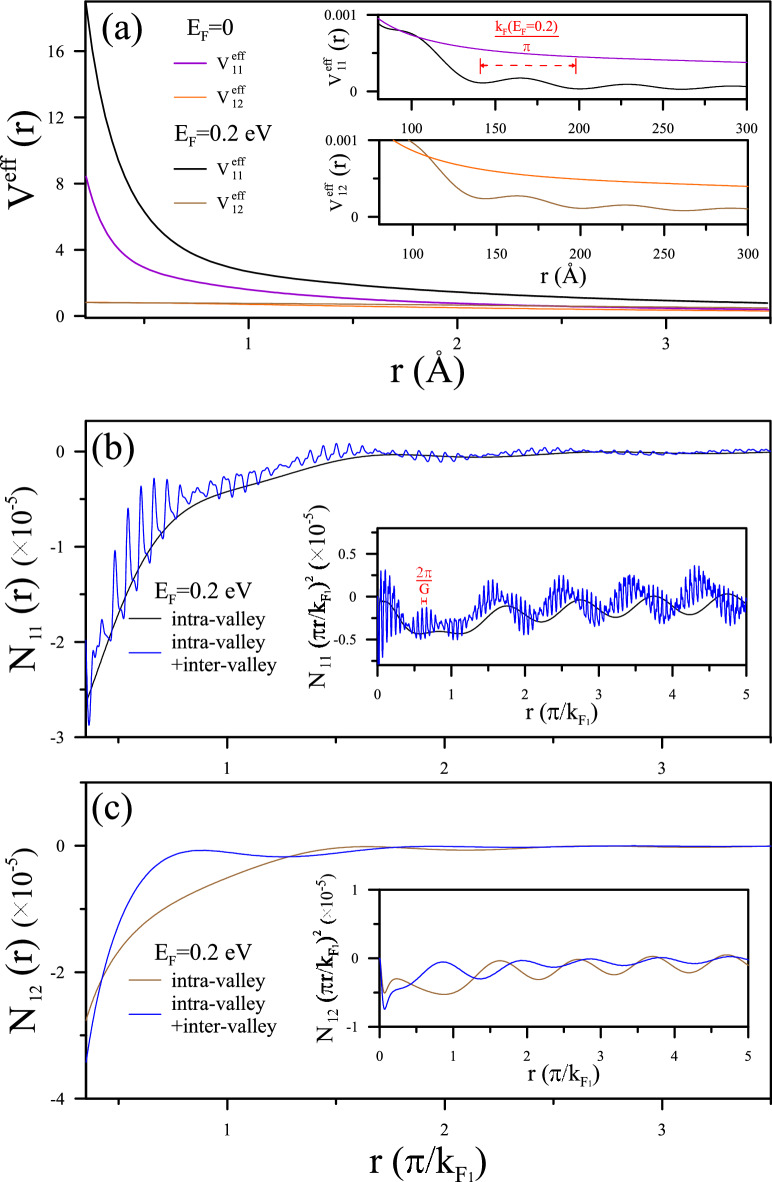
Comparative plot for AB-stacked BLG: The plot, akin to Fig. 4, is tailored for AB-stacked BLG, showcasing the effective potentials $V^{eff}_{11}$ and $V^{eff}_{12}$ at $E_{F}=0$ and $E_{F}=0.2$ eV. Additionally, it illustrates the induced charge densities $N^{in}{11}$ and $N^{in}{12}$ at $E_{F}=0.2$ eV, highlighting FOs characterized by a period of $\pi /k_{F}=\pi /k_{F_{1}}$.

**Figure 8:**
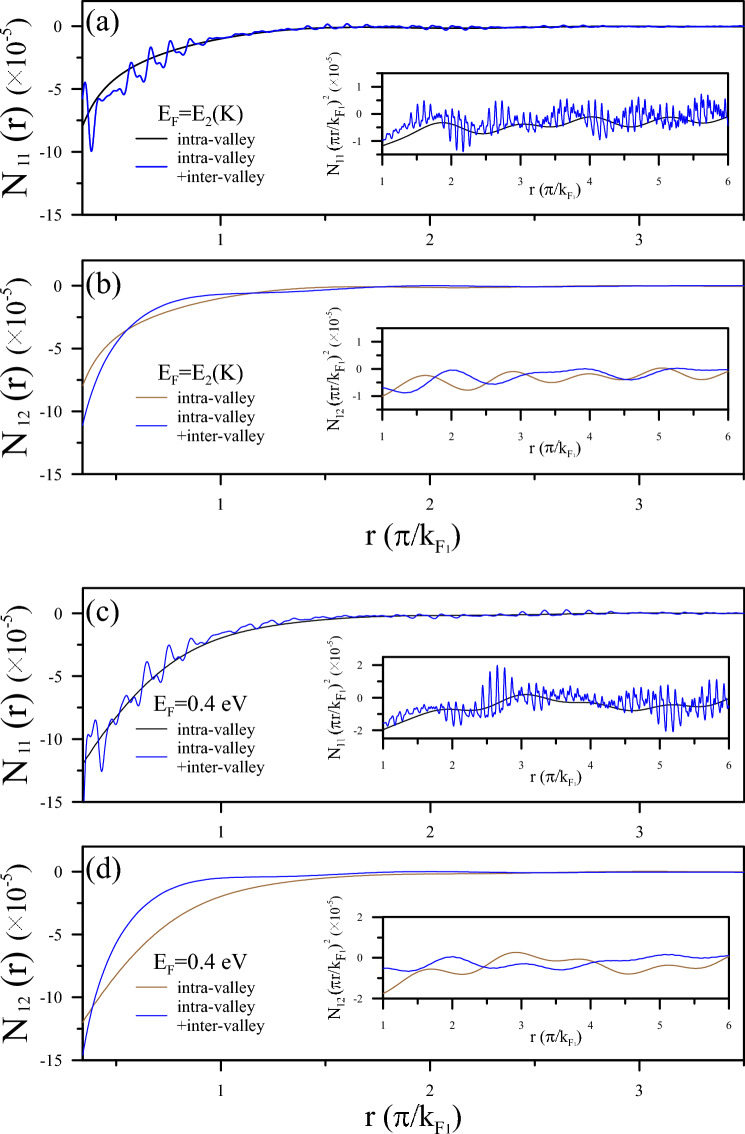
Comparative visualization of FOs in AB-stacked BLG: Figure provides a comparative view of $N_{11}$ and $N_{12}$ within AB-stacked BLG. It showcases the emergence of multiple FOs across different Fermi levels scaled by $k_{F_{1}}$.

Monolayer graphene forms a tightly bound hexagonal honeycomb lattice with a molecular bond length of $b=1.42$ ${\text{\AA }}$ (Fig. 1a). Primitive vectors $\mathbf {a_{1}}$ and $\mathbf {a_{2}}$ define the hexagonal Brillouin zone with non-equivalent Dirac points *K* and $K^{\prime }$ (Fig. 1b). The reciprocal lattice constant *G* is approximately 1.7 ${\text{\AA }}^{-1}$. The electronic states, represented by conical Dirac cones near each Dirac point, extend from the chemical potential with an energy width of $3\gamma _{0}$ and determine the Coulomb screening overall entire Brillouin zone.

In Fig. 1c, the variation of $\chi (q)$ with different doping levels is illustrated. Two types of scattering processes, intra-valley and inter-valley channels associated with *K*/$K^{\prime }$ points, are implicated in the Coulomb matrix and will be studied at different $E_{F}$ and $\phi$. $\chi (q)$ exhibits critical points ${q_c}$ (indicated by arrows), where discontinuous derivatives coincide with the Fermi-Dirac distribution. FOs originate from a singularity in the dielectric function at $q_{c}$, resulting from the Fermi-Dirac discontinuity at the Fermi surface contour. For undoped graphene, ${\chi (q)}$ (black curve) vanishes at the long-wavelength limit $q\longrightarrow 0$, indicating zero-gap semiconducting behavior at the Dirac point. It has been demonstrated that intra-valley channels exhibit an analytical function consistent within the linear band regime, while transitions between different valleys cause another set of derivative singularities at $q_{c}=G$ and $2G/\sqrt{3}$ with transitions occurring at $\phi =\pi /6$ and $\phi =0$, as indicated by the red hollow arrows in Fig. 1c.

With increasing electron doping, ${\chi (q\longrightarrow 0)}$ increases from zero to a finite value in the metallic system (dashed curve for $E_{F}=0.4$ eV). Intra-valley transitions further participate at $q_{c}=2k_{F}$ within each Dirac cone at *K* or $K^{\prime }$ points. In contrast, the inter-valley $q_{c}$ shifts by $\pm k_{F}$ (green hollow arrows) with variations in the Fermi surface contour from the Dirac point to a circular contour of radius $k_{F}$. The well-acknowledged nonanalyticity on the Fermi surface ($k_{F}$) significantly influences FO emergence, which at finite doping, is expected to show beating oscillations as a combination of the inter-valley and intra-valley channels.

When a charged impurity is implanted on the graphene sheet, $V^{eff}(q)$ derived from Eq. 6, satisfies RPA under $V^{ex}=v_q=2\pi e^2/q$. Performing the inverse FT into real space in Eq. 8 validates the intra-valley charge screening within the Dirac cone (Fig. 2a). In the undoped case (depicted by black curve), the long-distance potential is mainly described by non-oscillating behavior and the FOs are suppressed as shown in the inserts. In contrast, doped systems, such as the n-type monolayer graphene (illustrated by the blue curve for $E_{F}=0.4$ eV), exhibit metallic screening behavior. The Yukawa potential form ${exp(-r/r_c)/r}$ is presented within a small range close to the charged impurity at $r=0$. The screening length $r_{c}$ in the linear band regime decreases as the square root of the carrier density. This behavior is attributed to the $q\longrightarrow 0$ behavior of the screening effect, consistent with the Thomas-Fermi approximation for 2D electron gas (2DEG) observed in some graphene contexts^30^.

The screening of impurities is evident in the spatial distribution of induced charge density (Fig. 2b). The pristine system exhibits the charge neutrality level asymptotically. However, the doped system (e.g., at $E_{F}=0.4$ eV) fluctuates significantly, resulting in the redistribution of charges around the impurity to significantly screen the Coulomb field. Consistent with ${V^{eff}}(r)$ oscillation, the significant oscillating *N*(*r*) for distance away from $r_{c}$ manifests the intra-valley singularity at $q_{c}=2k_{F}$ with a $r^{-3}$ decay behavior shown in the insert. This oscillation behavior is clarified by the 2$k_{F}$-radius Fermi contour, proven by RPA^9^ and corroborated by experimental measurements of spatial standing waves^12,13,20^. It suggests that FOs play an equal role alongside Thomas-Fermi screening in the spatial behavior of quasiparticles due to the dielectric behaviors. This highlights the advantages of the RPA-based approach in concurrently addressing both short-range and long-range Coulomb scatterings as electron doping increases.

Regarding both intra-valley and inter-valley screenings, significant beating patterns in Fig. 2c emerge as charge fluctuations around the asymptotic level depicted in Fig. 2b. This phenomenon, absent in 2DEG^2^, is contributed by the *K* and $K^{\prime }$ valleys in graphene systems. Such beating patterns occur with electron doping in monolayer graphene, manifesting enhanced screening with two spatial modes of periods ${\pi /k_F}$ and $2\pi /G$. The former, classified as intra-valley scattering, serves as the main mode dominating static dielectric behavior. However, the latter from inter-valley between $K-K^{\prime }$ gives rise to beating due to large momentum transfer, which becomes crucial with an increase in conduction carriers, particularly for doped few-layer graphene. The structure of Fermi surface that cross few-layer subbands leads to multi-surface contours, thereby enriching and diversifying FOs with various stacking configurations such as AA and AB stacked graphene. These stacking-dependent properties of FOs offer an excellent opportunity to explore the screening phenomenon due to charge fluctuations in 2D materials.

### Unveiling Dirac characteristics in multimode FOs of AA-Stacked BLG

The AA-stacked bilayer configuration, known for its high symmetry and low-lying pairs of Dirac cones, provides an intriguing platform for the study of static Coulomb interactions. Commonly encountered in multilayer graphene synthesis processes, this configuration has attracted considerable theoretical and experimental interest due to its fascinating electronic properties and macroscopic stability^31^. This section aims to explore the effects of doping and stacking on static Coulomb excitations in the system, contributing to a deeper understanding of its complex and intriguing characteristics.

#### Electronic band structure and static Coulomb response

The highly symmetric AA-stacked BLG forms when one graphene sheet is precisely aligns directly above another. The geometric structure is depicted on the left side in Fig. 3a, where the tight-binding parameters $\alpha _{0}=2.569$ eV, $\alpha _{1}=0.361$ eV, and $\alpha _{3}=-0.032$ eV, along with the Hamiltonian (see^32^), define its hexagonal Brillouin zone. The electronic band structure features two vertically aligned Dirac cones at each *K* or $K^{\prime }$ Dirac point (depicted in black and brown), where critical momenta $q_{c}$ are associated with the representing static transitions by arrows.

The Hamiltonian matrix expanded around the Dirac points resembles renormalized monolayer-like sub-matrices, each possessing its spatial symmetry^33^. The Dirac-point energies, given by $2\cos [j\pi /(N_{l}+1)]\alpha _{1}$, where $j=1,2..N_{l}$. It results in $\pm E_{D}=\pm \alpha _{1}$ for BLG. The Fermi level in undoped BLG, evaluated as the average of the two Dirac point energies (defined as $E_{F}=0$), signifies an equal number of free electron-like carriers in Dirac cone of $1^{c,v}$ and hole-like carriers in that of $2^{c,v}$. The intra-band transitions within the two Dirac cones result in the same $q_{c}$ on the two Fermi surfaces, estimated as $q_{c}=2k_{F_{1}}(E_{F}=0)=2k_{F_{2}}(E_{F}=0)$ (depicted in red). As $E_{F}=E_{D}$, it diverges into two distinct values (illustrated in green), accompanied by an additional inter-pair $q_{c}=k_{F_{12}}(E_{F}=E_{D}$) (highlighted in cyan). Consequently, multimode FOs are expected to be triggered with diverse Fermi levels, leading to a variety of FO patterns.

The $2\times 2$ response tensor in AA-stacked BLG, expressed as a function of *q* derived from Eq. 5, encompasses intralayer and interlayer responses under static Coulomb interactions, specifically denoted as $\chi _{11}(q)$ and $\chi _{12}(q)$. Intra-valley scatterings within the same or different Dirac cones contribute to the primary modes of oscillation in the effective potential and induced charge density. AA-stacked BLG resembles a combination of two monolayer Dirac cones, each with its tunable carrier density associated with the Dirac points. While inter-valley scattering plays an equal role in intensity, it also introduces additional modes of FOs and collectively contributes to a beating pattern. Intriguingly, $\chi _{11}(q)$ and $\chi _{12}(q)$ exhibit distinct Coulomb responses associated with the two conical structures in the AA-stacking configuration. Those from intra-valley scattering, governing the main mode of FOs, are illustrated first. There exists a finite response in the long-wavelength limit at any Fermi level, indicating the semimetal characteristics’ screening ability of massless Dirac fermions.

In the undoped case, pristine free carriers contribute to a prominent characteristic at $q_{c}=2k_{F_{1}}(E_{F}=0)=2k_{F_{2}}(E_{F}=0)$, marked by the red arrow in Fig. 3b. The tilted step structure resulting from individual *K* or $K^{\prime }$ valleys is clearly visible near $q_{c}$, resembling monolayer behavior. However, upon doping, this structure undergoes splitting and shifting to both sides of their original position. Particularly, at $E_{F}=E_{D}$, $q_{c}=2k_{F_{2}}$ shrinks and disappears, while $q_{c}=2k_{F_{1}}$ shifts to twice the momentum, as shown by the dashed curve. In addition, the electronic scattering on the contour between two Fermi surfaces corresponds to another $q_{c}=k_{F_{12}}$. These significant changes in the critical momenta indicate intriguing intralayer screening effects of the impurities.

On the contrary, interlayer screening, represented by $\chi _{12}(q)$, manifests as a finite response only in the vicinity of these $q_{c}$ values, while remaining unresponsive to the external field otherwise. The $K/K^{\prime }$ transitions in Fig. 3c also induce steps at $q_{c}=2k_{F_{1}}(E_{F}=0)=2k_{F_{2}}(E_{F}=0)$ (red arrow). However, a local extreme is caused between two Dirac cones, specifically for $\chi _{12}(q)$ at $q_{c}=k_{F_{12}}$ (cyan), which is expected to dominate FOs on different layers with impurities. Although weak interlayer Coulomb matrices may render them ineligible, they could correspond to specific spatial oscillating periods within the material. These distinct responses between intralayer and interlayer screening are attributed to the multiple Dirac cone structures in the AA-stacking configuration^6^.

In Fig. 3d, e, $\chi _{11}(q)$ and $\chi _{12}(q)$ for the second $q_{c}$ region is depicted, representing inter-valley channels responsible for characteristic lengths on the order of 1/*G* ${\text{\AA }}$. With varying Fermi levels, these singular behaviors for the static response can manifest themselves with different periods and intensities of the beating FOs, revealing an intriguing phenomenon associated with the relationship between these $q_{c}$. Such oscillation modes would be mapped with periods of $\pi /k_{F_{1}}$, $\pi /k_{F_{2}}$, $\pi /k_{F_{12}}$ and $2\pi /G$ in the real space. These dielectric singularities need further experimental validations while the 2$k_{F}$-radius Fermi contour has been verified in AB-stacked BLG theoretically and experimentally^13,14^. Their significance on FOs should be evaluated relative to the overall behavior of the system, considering factors such as impurity position and magnitude. The combined effect of all singular polarizabilities may make the stacked system more likely to produce beating FOs.

#### Layer-dependent FOs due to charge impurity

The FOs on different layers in AA-stacked BLG can be assessed through the probing electrons on the relevant layers. In the scenario of a charged impurity introduced on the first graphene sheet, the perturbed potentials affecting the probing electrons associated with the two graphene sheets can be expressed as $V^{ex}_{11} = v_{q}$, $V^{ex}_{12} = v_q e^{-qd_0}$, $V^{ex}_{22} = V^{ex}_{11}e^{-qd_0}$, and $V^{ex}_{21} = V^{ex}_{22}e^{-qd_0}$ within the self-consistent approach. By substituting these values into the tensor equation in Eq. 6, along with the dielectric tensor, and performing the inverse FT in Eq. 8, we derive the total potential on one layer that encompasses contributions from charges in each layer.

As illustrated in Fig. 4a, at $E_{F}=0$, the long-distance decay of Coulomb potentials on the two graphene sheets demonstrates similar characteristics to metallic screening behaviors, but differs at short distances, especially regarding FO behaviors. ${V^{eff}_{11}} (r)$ exhibits a Yukawa potential form ${v_{q}\exp (-r/r_c)/r}$ within a short distance range close to the charged impurity, a feature being identified from intra-valley scattering. In the insert, strong and rapid FOs are demonstrated on the scale of $\pi /k_{F_{1}}(E_{F}=0)$. In contrast, ${V^{eff}_{12}}(r)$ exhibits screening of $1/\sqrt{d_{0}^{2}+r^{2}}$ and relatively weak FOs. This difference is observable in the distribution of the induced charge densities. The intralayer experiences stronger screening, while the distorted Dirac cones in BLG cause a divergence of the dielectric singularity at the critical momentums, which is relatively weak and slow.

In Fig. 4b, c, the induced charge density on the two graphene sheets, denoted as $N_{11}(r)$ and $N_{12}(r)$, is discussed. The majority of the induced charge is concentrated within the range of $r \le \pi /k_{F_{1}}$, effectively screening the external potential at short distances. This outcome manifests the intense intra-valley and equally significant inter-valley scattering in the first layer, contributing to the beating phenomenon of FOs. Considering that the oscillation amplitude should be proportional to the external Coulomb interaction and Coulomb matrix, the beating behavior is distinctly visible only for $N_{11}(r)$ but remains unobserved in $N_{12}(r)$ even as an amount of charge accumulates closer to the impurity. It should be noticed that the $1/r^2$ decay evident in the inserts signifies a slower rate of decrease at long distances. This corresponds to a more gradual reduction in the screened potential observed in Fig. 4a. This feature implies an extended influence of the charged system over a greater distance than a monolayer. In other words, while $N_{11}(r)$ originates from intra-pair transitions and resembles the screening effect of monolayer Dirac quasi-particles, $N_{12}(r)$ is attributed to inter-pair transitions exclusively persisted in AA-stacked BLG.

Also suggested are tunable multimode oscillations due to the relevant singular responses of the two-fold Dirac cones under varying dopings. At $E_{F}=E_{D}$, three possible Fermi contour transitions may participate in the FOs. For $N_{11}(r)$ (Fig. 5a), the periods manifest scaling with intra-valley channels of 2$k_{F_{1}}$ and are modulated by beating patterns from inter-valley ones, as explained by the behaviors of $\chi _{11}(q)$ in Fig. 4b. The dominance of intra-pair transitions is specifically clarified by the FO period of $k_{F_{1}}$ and reveals the massless Dirac cone nature. On the other hand, in Fig. 5b, the interlayer FO, $N_{12}(r)$, is governed by inter-valley channels, specifically illustrated by $q_{c}=k_{F_{12}}$ in $\chi _{12}(q)$, as depicted in Fig. 3d. The main mode of FOs is twice in $N_{12}(r)$ than in $N_{11}(r)$. Consequently, the different FO behaviors on the two graphene sheets are consistent with the exploration of the layer-indexed response functions. Complex FOs can arise from the involvement of multiple oscillating modes originating from the two Fermi surface contours. The collective impact of all singular polarizabilities may increase the likelihood of generating FOs, as evidenced by the periods, intensities, and beating patterns of the oscillation characteristics. These effects can be verified through STM^20–23^.

### Decoding FOs in AB-stacked BLG: unraveling precision in layer alignment

The AB-stacked (Bernal) configuration of BLG stands as a cornerstone in the realm of two-dimensional materials, showcasing unique electronic properties and structural intricacies. This bilayer arrangement forms an inversion symmetric pattern (left insert Fig. 6a). The A atoms of the bottom layer (red color) precisely align directly beneath B atoms on the top layer, while B (A) atoms on the bottom (top) situated at the hexagon centers of the top (bottom) layer. This inversion symmetry signifies a critical aspect of BLG’s behavior. In this context, the interplay of electronic behavior, band structure, and intralayer and interlayer coupling is elegantly described by a ${4\times 4}$ Hermitian matrix in the 2$p_z$-based Bloch functions. These fundamental attributes, coupled with atomic hopping integrals, denoted as $\gamma _{0},..., \gamma _{4}$ (indicated in the insert), as modeled by the Slonczewski-Weiss-McClure (SWMcC) approach^32,34^, provide a robust framework for exploring BLG’s electronic^35^, optical^36^, and transport characteristics^37^.

#### Electronic band structure and static Coulomb response

The band structure of AB-stacked BLG features two pairs of parabolic energy bands, primarily influenced by the ${2p_z}$ orbitals within a primitive unit cell comprising four carbon atoms. Close to the *K*/$K^{\prime }$ points, one pair of parabolic bands, forming a valence-conduction band crossing at $E_{F}=0$, characterizes it as a zero-gap semimetal (depicted by $1^{c,v}$). Simultaneously, the second pair experiences an energy shift of $\gamma _{1}$ away from $E_{F}=0$ (depicted by $2^{c,v}$), with the band edges at K denoted as $E^{2^{c,v}}(K)$^32^.

The two tensor components of the static polarization function, denoted as $\chi _{11}(q)$ and $\chi _{12}(q)$ in Eq. (4), are computed for AB-stacked BLG. The calculation is predominantly influenced by the two pairs of parabolic bands, $1^{c,v}$ and $2^{c,v}$, situated at the K or K$^{\prime }$ point, as illustrated in Fig. 6b–e. The semimetal response is comparatively weaker in pristine AB-stacked BLG (black curves) than in AA-stacked BLG, a result of the rare pristine carrier density. Specifically, the intra-valley channels, illustrated in Fig. 6b, c, predominantly influence the low-*q* behavior and impact the oscillating characteristics of the effective potential and induced charge density. It is inferred from the singularities in $\chi _{11}(q)$ and $\chi _{12}(q)$ that the number and value of $q_{c}$ depend on individual Fermi contours and satisfy the selection rule $1^{c,v}\leftrightarrow 1^{c,v}$ and $2^{c,v}\leftrightarrow 2^{c,v}$. Consequently, there exists one critical $q_{c}$ for transitions within the same valley when the Fermi level is lower than the band edge $E^{2^{c}}(K)$, while inter-Fermi contour transitions are suppressed under Coulomb interactions^10^.

Overall, it is evident that the two independent response components characterize the screening effects with FOs manifested by multiple critical momenta $q_{c}$ around 2$k_{F_{1}}$ and 2$k_{F_{2}}$, respectively, from $1^{c,v}\leftrightarrow 1^{c,v}$ and $2^{c,v}\leftrightarrow 2^{c,v}$. These intra-pair channels conspicuously affect FOs, particularly in scenarios with different geometries of Fermi contours within the $1^{c,v}$ and $2^{c,v}$ subbands. On the other hand, inter-valley response, leading to additional beating feature modes in the order of 1/*G*, is highlighted in Fig. 6d, e, exhibiting different features than those in AA-stacked BLG due to the selection rule. The screening phenomenon is more controllable through Fermi energy in the AB-stacking configuration, as expected to be evidenced by the characteristics of FOs, which are diversified by the stacking configurations.

#### Layer-dependent FOs due to charge impurity

The AB-stacked BLG presents intriguing static screening phenomena in response to charge impurity, influenced by its unique band structure and response tensor. The layer-dependent screening effect on each layer in AB-stacked BLG, with the relative position of a single impurity on the first layer, is demonstrated in terms of effective Coulomb potential and induced charge density. As illustrated in Fig. 7a, the enhanced screening effects due to conduction electrons are observed as $E_{F}$ increases from 0 to 0.2 eV, accounting for parabolic bands that are distinct from AA-stacked graphenes. At $E_{F}=0.2$ eV, a Yukawa potential decay factor ${\exp (-r/r_c)}$ is demonstrated on ${V^{eff}_{11}}(r)$, but not on ${V^{eff}_{12}}(r)$, in contrast to the slow 1/*r* decay for the undoped case at $E_{F}=0$. This feature is characteristic of multilayer graphene screening a single impurity immersed in it. Observing the FOs of the induced charge density is particularly important in BLG. The well-behaved FOs on the two graphene layers are evidenced by their display with a period of $k_{F_{1}}/\pi$ at $E_{F}=0.2$ eV for long distances, depicted in Fig. 7b, c, while also revealing a long-distance $1/r^{2}$ decay on both layers in the inserts. Meanwhile, identified as a bilayer FO characteristic, a beating pattern is demonstrated in the former, and a phase shift is caused in the latter as a result of inter-valley scattering.

However, the multimode FOs in AB stacking are very different from the AA stacking. The induced charge density oscillates solely in modes corresponding to specific Fermi surface contours under appropriate electron doping, namely, intra-pair channels $q_{c}=2k_{F_{1}}$ and $q_{c}=2k_{F_{2}}$. This phenomenon arises due to the AB stacking configuration, where inter-pair singularities are inhibited by the special selection rule for transitions of Fermi states. For example, in Fig. 8a, b, for $E_{F}$ just across the band edge of subband $2^{c}$, i.e., $E_{F}=E_{2}(K)$, the single mode is $q_{c}=2k_{F_{1}}$, and the electronic scattering is manifested. As explained above in the response functions, the FOs reflect the intra-pair mode, which relatively causes the singularity in the dielectric function to become prominent, so the FOs are also the same. As a consequence, the Coulomb field screening effect at $E_{F}=0.4$ eV in Fig. 8c, d can be proven by a combination of both the two intra-pair modes for the same reason.

In brief, the static screening effect in few-layer graphene is contingent upon both the quantity and placement of impurities. Interlayer interference gives rise to quasiparticle scattering near the Fermi surface, as evidenced by the overall Friedel potential. These dependencies extend to the distinct electronic structures and wave functions characteristic of AA and AB stacking configurations. In particular, interlayer screening in BLG signifies diminished Coulomb interaction and a relatively coupled electron nature between layers. The interlayer interference contributes significantly to the electronic characteristics and overall FO, thereby playing a pivotal role in shaping the behavior of Fermi surface quasiparticles and their associated physical properties.

It should be noticed that the period and amplitude of the beating patterns come from the multiple Fermi circles. Previous studies have shown the theoretical and experimental validations of the STM maps due to the intra- and inter-valley scattering in AB-stacked BLG^13,14^. While the intra-valley period has been verified in AB-stacked BLG, the $r^{2}$ decay factor observed in experiments requires further refinement^22^. In addition, the inter-valley scattering can be verified by the beating patterns, as demonstrated by valley scattering on multiple Fermi circles by graphene armchair and zigzag edges^20^. The observed standing waves reflect the scattering intensity, the geometry of the Fermi surface, and the STM map, providing experimental validation for this theoretical models. This approach not only complements existing literature but also extends it by providing a more detailed understanding of the dielectric singularity due to multiple Fermi circles.

FOs emerge as intricate quantum phenomena with the potential to unravel the subatomic mysteries within 2D materials. Following the crucial role played by advanced techniques like high-resolution STM in uncovering real-space charge dynamics^38^, these approaches have further illuminated various phenomena in the charge density distribution influenced by Coulomb screenings. For, example, high-resolution STM observations have revealed dipole screening in semiconductors^39^, long-range FOs in metals^40^, ${1/r^2}$ decay^41^ and many-body effects in 2DEG^42^, and quasiparticle interference in graphene quantum dots^21^. Moreover, recent STM surface morphology has successfully clarified quantum topologies in graphene^17–19^. Expanding our insights to graphene measurements, STM’s capability to provide valuable insights into the charge fluctuations around defects or impurities becomes particularly significant. Notably, graphene quantum dots display novel quasi-bound states under electrostatic confinement governed by Klein tunneling^21^. These quasiparticles in graphene systems can lead to distinct backscattering and unique FOs, which can be further enriched by the varying confinements associated with different stacking, configurations and boundaries. This methodology serves as a powerful tool for validating FOs in real-space, providing direct confirmation of the approach across various contexts.

### Concluding remarks

This article extensively explores FOs in graphene, with a specific focus on monolayer graphene, as well as AA- and AB-stacked BLG. The study unveils layer-specific attributes, placing emphasis on stacking-dependent multimode behaviors. The model, utilizing layer-based tight-binding functions, unifies critical factors such as orbital hybridizations and Coulomb interactions. It enables a self-consistent approach to calculate induced potentials on individual layers and systematically investigate dominant electron scattering mechanisms.

FOs, rooted in Fermi contour geometry, are explored for their dependence on electron doping effects, valley-dependent charge rearrangements, altered screening, and diverse oscillations. Understanding screening phenomena is employed to comprehend Coulomb scattering properties, accounting for the electronic band structure and wave functions. The exploration of metallic screening reveals intricate characteristics, including Yukawa potential length, impurity-related charge fluctuations, single/multi-mode FOs, beating screening, and non-oscillatory interlayer behavior within graphene systems.

Monolayer graphene exhibits beating FOs due to cooperative behaviors from inter-valley and intra-valley channels, while BLG demonstrates intricate multimode FOs attributed to weakened screening effects from interlayer interactions. The influence of charged impurities on the graphene sheet elucidates screening behavior within individual pairs of bands or different bands, resulting in distinct behaviors for AA- and AB-stacked BLG. The unique band structures in AA- and AB-stacked BLG, specifically massless multiple Dirac and massive Dirac fermions, characterize a distinct pattern in the static polarization function tensor and its role in screening effects. The exploration of beating FOs aims to comprehend varying behaviors for AA- and AB-stacked BLG. Furthermore, the screening phenomenon is more controllable through Fermi energy, leading to diversified FOs. This signifies the modulation of FOs on different layers concerning impurity in systems, with potential insights through STM observations on materials to unveil real-space charge dynamics.

In summary, this comprehensive exploration of FOs in graphene systems provides a nuanced understanding of screening phenomena, paving the way for further research and applications in the field. The study reveals distinctive layer-dependent static screening behaviors in BLG configurations. The versatility of high-resolution STM serves as a valuable tool for confirming theoretical predictions. The amalgamation of theoretical insights and experimental validations significantly contributes to advancing knowledge in condensed matter physics.

## Data Availability

This article includes all data produced and analyzed during this investigation.
